# Subtilisin-like proteases in plant–pathogen recognition and immune priming: a perspective

**DOI:** 10.3389/fpls.2014.00739

**Published:** 2014-12-19

**Authors:** Andreia Figueiredo, Filipa Monteiro, Mónica Sebastiana

**Affiliations:** Plant Systems Biology Lab, Center for Biodiversity, Functional and Integrative Genomics, Science Faculty of Lisbon University, Lisbon, Portugal

**Keywords:** plant resistance, enhanced immunity, programmed cell death, subtilase, protease, crop improvement

## Abstract

Subtilisin-like proteases (subtilases) are serine proteases that fulfill highly specific functions in plant development and signaling cascades. Over the last decades, it has been shown that several subtilases are specifically induced following pathogen infection and very recently an *Arabidopsis* subtilase (SBT3.3) was hypothesized to function as a receptor located in the plasma membrane activating downstream immune signaling processes. Despite their prevalence and potential relevance in the regulation of plant defense mechanisms and crop improvement, our current understanding of subtilase function is still very limited. In this perspective article, we overview the current status and highlight the involvement of subtilases in pathogen recognition and immune priming.

## LINKING PROTEOLYSIS TO PATHOGEN RECOGNITION: THE ROLE OF PLANT SUBTILISIN-LIKE PROTEASES

Proteolysis is fundamental for the normal functioning of multicellular organisms and plays key roles in a variety of processes such as development, physiology, defense and stress responses, and adaptation to the changing environment. The serine proteases are one of the best characterized groups of proteolytic enzymes in higher organisms.

Subtilisin-like proteases (subtilases) are serine proteases characterized by a catalytic triad of the three amino acids, aspartate, histidine, and serine (Dodson and Wlodawer, 1998). According to the MEROPS classification (http://merops.sanger.ac.uk), Eukaryotic subtilases constitute the S8 family within the SB clan of serine proteases (Rawlings et al., 2006). Plant subtilases correspond to S8A subtilisin subfamily and form an extensive group of enzymes, whereas S8B (kexin-type) proteins appear to be absent from plants (Tripathi and Sowdhamini, 2006). Subtilases are especially abundant in plants, with 63 genes known in the *Oryza sativa*, 56 genes in *Arabidopsis thaliana* and at least 15 in *Lycopersicon esculentum* genomes (Meichtry et al., 1999; Rautengarten et al., 2005; Tripathi and Sowdhamini, 2006).

**FIGURE 1:**
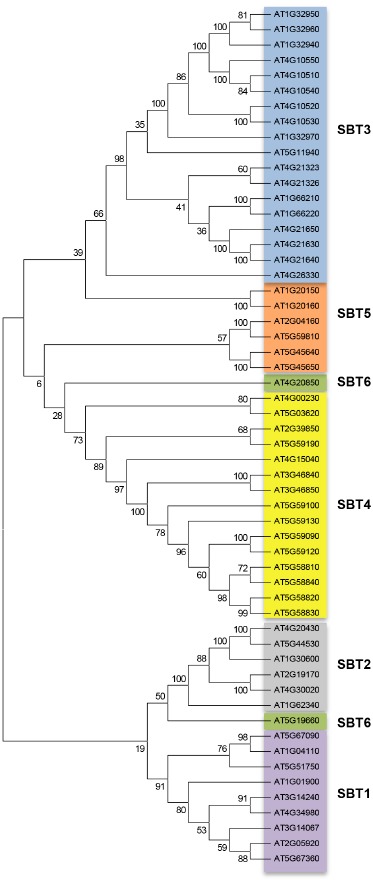
**Bootstrapped consensus neighbor-joining (NJ) tree generated from the alignment of the 56 protein sequences annotated as AtSBT, using Mega 6.06 software.** SBT1, SBT2, SBT3, SBT4, SBT5, and SBT6 are highlighted with different colors with NJ tree demonstrating that each subfamily is well resolved phylogenetically (bootstrap values >50).

The knowledge of the phylogenetic relationships of subtilase genes may help to unravel their basic functions based on the annotation of the orthologous sequences. When analyzing the 56 *Arabidopsis* sequences (Figure 1), based on sequence similarity, we were able to discriminate the six subfamilies already described by Rautengarten et al. (2005). These authors have shown that within the 56 *Arabidopsis* subtilase genes, 55 presented the characteristic conserved motifs (S8 domain and the aspartate, histidine, and serine residues) and 53 presented the protease-associated (PA) domain, associated to the determination of substrate specificities and protein–protein interactions (Rautengarten et al., 2005). Our blast2GO annotation of the 56 *Arabidopsis* sequences showed that some biological processes and functions were common to the sequences within each subfamily: SBT3 was associated to “detection of biotic stimulus and detection of external stimulus”; SBT4 was associated to “petal and stamen development” and SBT5 was associated to “oxidoreductase activity.” Although several studies have been made in order to characterize plant subtilases (Tornero et al., 1997; Berger and Altmann, 2000; Schaller, 2004; Liu et al., 2009; Budic et al., 2013; Ramirez et al., 2013) the function of the majority of them remains unknown.

In plant–pathogen interactions, the first evidence for the importance of plant subtilisin-like proteins was reported in tomato, where expression of the subtilases P69B and P69C was induced following pathogen attack and salicylic acid (SA) application (Tornero et al., 1996a; Jorda et al., 1999). Furthermore it was shown that these subtilases are glycosylated and secreted to the plant extracellular matrix (ECM) where they accumulate (Yamagata et al., 1994; Tornero et al., 1996a,b, 1997; Siezen and Leunissen, 1997; Taylor et al., 1997). Considering that ECM is where the first host–pathogen interaction, recognition and signaling events take place (Dixon and Lamb, 1990), the accumulation of subtilases in plant ECM may account for an important role during pathogenesis. It was also shown that P69C specifically processes a LRP protein in disease tomato plants that belongs to the extracellular leucine-rich repeat (LRR) family of proteins. LRR proteins mediate molecular recognition and/or interaction processes in the ECM of eukaryotic cells to initiate different signaling processes (Tornero et al., 1996b). Recently, when comparing resistant and susceptible grapevine genotypes, a subtilisin-like protein sharing sequence similarity with the tomato P69C was shown to be constitutively expressed in the resistant genotype, its expression being induced after *Plasmopara viticola* inoculation (Figueiredo et al., 2008, 2012; Monteiro et al., 2013).

## IMMUNE RESPONSES AND PROGRAMMED CELL DEATH

Having in mind that plants face a constant array of invading microorganisms and that only a small percentage of plant–pathogen interactions leads to successful disease development, plants have first to perceive the pathogen and then to activate an innate immune system in a timely, accurate, and effective manner. Perception initially involves the detection of broadly conserved molecules, known as microbe- or pathogen-associated molecular patterns (MAMPs or PAMPs) by plasma membrane proteins known as pattern recognition receptors (PRRs). PAMP-triggered immunity (PTI) constitutes a front-line pattern-triggered immune response that must be overcome by microorganisms for successful colonization of plant tissues (Jones and Dangl, 2006). PTI is characterized by the rapid production of reactive oxygen species (ROS), activation of signaling cascades and by an overall transcriptional reprogramming favoring defense (Moore et al., 2011). A second intracellular class of immune receptors is activated via recognition of pathogen effectors, resulting in effector-triggered immunity (ETI). ETI is mediated by the nucleotide-binding domain leucine rich repeat (NB-LRR) disease resistance proteins and is often manifested as a hypersensitive response (HR) associated with rapid cell death, production of ROS and SA, and expression of defense-related genes (Jones and Dangl, 2006). Apart from pathogen-derived elicitors that can activate the plant innate immune response, plant endogenous elicitors that trigger or amplify the innate immune response have also been identified (Ryan and Pearce, 2003; Huffaker et al., 2006; Huffaker and Ryan, 2007; Pearce et al., 2010). Of those, a 12 amino acid peptide from soybean derived from an extracellular subtilase(Glyma18g48580) was shown to activate defense-related genes, leading to the hypothesis that, upon pathogen attack, this endogenous peptide would be available for receptor binding and initiation of defense signaling pathways (Pearce et al., 2010; Yamaguchi et al., 2011).

Another interesting feature of subtilisin-like proteins, which was recently reviewed by Vartapetian et al. (2011), is their involvement in plant programmed cell death (PCD). Cell death has a central role in innate immune responses in both plants and animals (Coll et al., 2011). Pathogen recognition leads to inhibition of pathogen growth, which is often, but not always, accompanied in plants by the triggering of the HR, a form of PCD localized at the site of attempted pathogen invasion. Current data indicate that the role played by caspases in animal PCD is taken, at least in part, by some subgroups of plant subtilisin-like proteases namely by phytaspases (Chichkova et al., 2010; Vartapetian et al., 2011). In 2012, it was shown that using serine inhibitors partially inhibited the overall activation of PCD and thereby changed the level of susceptibility of grapevine toward the oomycete *P. viticola* (Gindro et al., 2012). In plant–oomycete interaction, the death stimuli may be triggered by the pathogen effectors and, among these protein effectors, protease inhibitors are crucial for successful suppression of plant defenses (van der Hoorn and Jones, 2004; van der Hoorn, 2008). It has been suggested that the secretome of *P. viticola* could inhibit the caspase-like proteases of grapevine susceptible varieties, thereby inhibiting the plant’s normal defense reaction. By contrast, resistant grapevine varieties would possess caspase-like proteases that are not recognized by the secretome of *P. viticola* due to slight structural modifications of the protein patterns of these cultivars. In this case, plant defense mechanisms would continue to operate, producing fatal consequences for the pathogen and restricting its development (Gindro et al., 2012).

So, up to this point, we have shown that several subtilisin-like proteases are associated to plant–pathogen resistance, that they are secreted to the ECM and may exert important functions both in pathogen recognition and initiation of signaling cascades leading to the activation of defense-related genes and that some sub-groups of the subtilase family play an important role in PCD. However, in plant–pathogen interactions one very interesting feature of subtilases was only recently identified, and may be linked to immune priming events.

## SUBTILASES LINKED TO IMMUNE PRIMING IN PLANTS

Plants are capable of establishing immune responses that are highly specific, with restricted self-reactivity, and that often generate a lifelong “memory” of the encountered pathogens. So, different immune strategies are used, being particularly relevant the enhancement of the potential to mount defense responses to subsequent infections where plants respond to much lower levels of a pathogenic stimulus in a more rapid and robust manner—a priming phenomenon (Beckers and Conrath, 2007). Even though the molecular mechanisms of priming remain elusive, it was proposed that cell priming involves accumulation of inactive cellular proteins that play an important role in cellular signal amplification (Bruce et al., 2007; Conrath, 2011). Subsequent exposure to biotic or abiotic stress could activate these dormant signaling proteins, thereby initiating signal amplification and lead to more rapid and robust activation of defense, immunity, and stress tolerance.

The study by Ramirez et al. (2013) identified the *Arabidopsis SBT3.3* gene, encoding a serine protease homologous to the tomato P69C subtilase. Similarly to the tomato P69C, *Arabidopsis* SBT3.3 protein may be linked to pathogen recognition and activation of signaling processes. It was shown that the expression of SBT3.3 is rapidly demanded during the activation of innate immunity preceding the activation of SA responsive genes and responding very rapidly to H_2_O_2_, a common ROS species generated very early during PAMP recognition by PRR leading to activation of innate immune responses. SBT3.3 substrate was not yet identified but it was hypothesized that it may process an extracellular domain (ectodomain) of a larger protein, likely functioning as a receptor located in the plasma membrane. After proteolytic shedding of the ectodomain, the receptor could become activated and initiate a downstream immune signaling process, similarly to what was described in animals (Ramirez et al., 2013). It is also hypothesized that after initiation of the signaling process, a positive feedback loop circuit would maintain the SBT3.3 expression. Maintenance of this expression threshold level should be sufficient to keep cells in a sustained sensitized mode (Ramirez et al., 2013). This expression pattern would consequently be the basis to explain the memory-based characteristics of priming and induced resistance. Future challenges rely on the identification of disease resistance subtilases target substrate and in the elucidation of their participation in the immune priming activation

## CONCLUSION

One of modern’s agriculture demands is to enhance harvest yields per acreage while reducing pre-harvest and post-harvest losses caused by pathogens. Modern pest management strategies in crop plants include classical and molecular marker-based resistance breeding, genetic engineering of plant immunity and the use of chemicals as pesticides or strengtheners of plant health. Here, we have highlighted the involvement of plant subtilisin-like proteins in both disease resistance and priming events. Despite all the recent advances on subtilase characterization, very little is known about their functions and substrates. Future research efforts have to be made to characterize these proteases in a broad spectrum of crop plants, to define their substrates and to prove their involvement in plant immunity so that subtilases may be considered has promising genomic tools to engineer durable, broad-spectrum plant disease resistance.

### Conflict of Interest Statement

The authors declare that the research was conducted in the absence of any commercial or financial relationships that could be construed as a potential conflict of interest.
